# Analysis of the Rehabilitation Efficacy and Nutritional Status of Patients After Endoscopic Radical Thyroidectomy by Fast Track Surgery Based on Nutritional Support

**DOI:** 10.3389/fsurg.2022.897616

**Published:** 2022-05-02

**Authors:** Fang Qu, Hongxia Bu, Liu Yang, Hui Liu, Chaoying Xie

**Affiliations:** ^1^Minimally Invasive Surgery Center of the First Hospital of Changsha, Changsha, China; ^2^Outpatient Office, The First Hospital of Changsha, Changsha, China

**Keywords:** lumpectomy, radical thyroidectomy, nutritional support, fast track surgery, rehabilitation outcome, nutritional status

## Abstract

**Objective:**

To investigate and analyze the effect of fast track surgery (FTS) based on nutritional support on the improvement of rehabilitation efficacy and nutritional status of patients after radical lumpectomy for thyroid cancer.

**Methods:**

Eighty-six patients admitted to our hospital for radical lumpectomy for thyroid cancer between April 2018 and April 2021 were selected, of which 40 patients admitted between April 2018 and April 2019 were included in the control group with conventional perioperative care. Forty-six patients admitted between May 2019 and April 2021 were included in the trial group with FTS care based on nutritional support. The two groups of patients were compared in terms of postoperative feeding time, length of stay, time out of bed, VAS scores, albumin (ALB), total protein (TP) and prealbumin (PA) levels, negative emotions [Mental Health Test Questionnaire (DCL-90)], quality of life [General Quality of Life Inventory (GQOLI-74)] and complication rates.

**Results:**

The patients in the trial group had shorter feeding time, hospitalization time and time out of bed than the control group (*P* < 0.05). After the intervention, ALB, TP and PA levels were higher in the trial group than in the control group vs. preoperatively (*P* < 0.05); VAS scores in the trial group were lower than VAS scores in the control group during the same period (*P* < 0.05). The postoperative DCL-90 scores of the trial group were lower than those of the control group (*P* < 0.05); the GQOLI-74 scores and total scores of the trial group were higher than those of the control group at the 3-month postoperative follow-up (*P* < 0.05). The overall incidence of complications such as hoarseness, choking on water, hand and foot numbness, wound infection, and hypocalemia was lower in the trial group than in the control group (*P* < 0.05).

**Conclusion:**

The implementation of FTS care based on nutritional support for patients after endoscopic radical thyroidectomyr can effectively improve the postoperative recovery and reduce their pain level, as well as help improve their nutritional status, negative emotions and improve their quality of life, which is worth promoting.

## Research Background

Thyroid cancer (TC) is a malignant tumor occurring in the thyroid area, with a low incidence rate, but due to its special location, the disease is more serious after its onset. TC is a common malignant tumor in endocrine, and surveys show that the incidence of TC in our population is increasing year by year, and it is mostly seen in young and middle-aged women (1, 2). Currently, the most effective way to treat thyroid cancer is surgical treatment, but conventional thyroid surgery leaves a long surgical incision scar on the neck, which affects the aesthetics and causes great psychological stress to patients (3). With the development of modern society, people have higher and higher requirements for aesthetic results after surgery. After lumpectomy thyroid surgery has been gradually carried out in China, it is popular among patients, especially female patients, for its unique cosmetic and minimally invasive effects. Although endoscopic radical thyroidectomy is a minimally invasive surgery with little damage to the patient's organism, postoperative patients are often complicated by anemia, diabetes mellitus, hypertension, coagulation dysfunction and electrolyte disorders due to the physiological tissue characteristics of cancer, which seriously affect the nutritional status and prognosis of patients (4, 5). Most patients have a slow recovery after surgery due to pain and complications, and some patients have low understanding of their disease and treatment, so they have greater doubts about the safety of treatment and postoperative life, which leads to a certain degree of negative emotions (6, 7). In this regard, the clinical should strengthen the effectiveness of perioperative nutritional support and nursing interventions.

Fast track surgery (FTS) refers to an innovative concept that perfectly combines the diagnosis and treatment plans with the latest research evidence during the perioperative period to promote the early recovery of organ function after surgery, including the latest minimally invasive techniques, anesthesia, postoperative analgesia, early postoperative enteral nutrition, early bed mobility, active rehabilitation exercises and psychological care (8, 9). Gradually applied in the perioperative period of thyroid cancer and has achieved results in relieving patient anxiety and pain, reducing postoperative complications, decreasing stress reactions, earlier postoperative bedtime, shortening hospital stay, improving quality of life, and improving quality of care and satisfaction (10, 11). We selected patients treated with radical lumpectomy for thyroid cancer and applied NRS 2002 for nutritional risk screening and daring to investigate the effect of the FTS model based on nutritional support on the recovery outcome and nutritional status of patients treated with radical lumpectomy for thyroid cancer.

## Object and Methods

### Study Subjects

Eighty-six patients admitted to our hospital who underwent lumpectomy for radical thyroid cancer between April 2018 and December 2020 were selected and their clinical data were collected.

### Inclusion Criteria

I. Diagnosis of thyroid cancer confirmed by puncture biopsy; II. Those who met the indications for radical thyroid cancer surgery and agreed to take surgical treatment; III. No distant metastasis on preoperative imaging, no combination of other malignant tumors, and no contraindication to relevant surgery; patients with cosmetic intention and no history of neck surgery or radiotherapy; post-hospital nutrition risk screening tool (NRS) 2002 score ≥ 3 points.

### Exclusion Criteria

I. Those with speech impairment and impaired consciousness, unable to communicate basically; II. Combined with other thyroid diseases; III. Contraindications related to surgery and anesthesia; IV. Comorbid with other tumors.

Among them, 40 patients admitted from April 2018 to April 2019 with conventional perioperative care were included in the control group. Fort-six patients admitted between May 2019 and April 2021 with rapid rehabilitation surgical care based on nutritional support were included in the experimental group.

### Data Collection

Basic data such as gender, age, tumor diameter, lesion site, body weight, preoperative albumin, total protein and prealbumin levels, nutritional score, operative time were collected and compared between the two groups, and the analysis showed that the differences were not significant and the bases were comparable (*P* > 0.05) (Table 1).

**Table 1:** Comparison of the base of 2 groups (Mean, SD; %).

**Information**		**Control group** **(*n* = 40)**	**Trail group** **(*n* = 46)**	***t* or χ^2^ value**	***P-*value**
Gender	Male	10 (25.00)	11 (23.91)	0.014	0.907
	Female	30 (76.00)	35 (76.09)		
Age (years)		42.57 ± 4.51	42.26 ± 4.65	0.313	0.755
Tumor diameter (cm)		1.50 ± 0.40	1.40 ± 0.50	1.01	0.314
Body weight (kg)		56.13 ± 7.20	56.50 ± 6.89	0.243	0.808
Lesion site	Left side	18 (45.00)	20 (43.48)	0.020	0.887
	Right side	22 (55.00)	26 (56.52)		
Albumin (g/L)		37.24 ± 3.21	38.16 ± 3.37	1.291	0.200
Total protein (g/L)		67.22 ± 11.17	68.45 ± 12.10	0.487	0.627
Prealbumin (mg/L)		250.50 ± 30.89	252.37 ± 33.14	0.269	0.788
NRS 2002 score (points)		4.10 ± 0.50	4.06 ± 0.45	0.390	0.697
Operating time (min)		85.64 ± 10.56	83.18 ± 11.25	1.040	0.301

## Intervention Methods

### Control Group

Patients were perfected with preoperative routine examination and completed preoperative preparation. Combined anesthesia was used, a catheter was left in place before surgery, and patients' vital signs were closely monitored intraoperatively. Postoperatively, they gradually received rehabilitation training, and analgesic drugs were given when pain occurred during exercise. Patients in the control group ordered the regular package from the hospital nutrition cafeteria and were not given standard nutritional support treatment, while the rest were given routine perioperative care.

### Trial Group

FTS care based on nutritional support was used, and nutritional support was individualized according to the patient's physical condition.

#### Preoperative Support

Application of parenteral nutrition support or enteral nutrition for more than 5 days was considered to be the use of nutritional support, nutritional support regimen for oral nutrition preparations and tube feeding, parenteral nutrition support regimen includes intravenous infusion of amino acids, glucose and fat emulsion. Patients without gastrointestinal motility disorders were fasted from solid diet 6 h before surgery, fasted from clear liquid 2 h before surgery, and given sugar water for energy supplement 2 h before surgery. Actively educate patients about the rapid recovery program to obtain better cooperation from patients. Before the operation, we perfected the routine examination and entered the operating room after completing the preoperative preparation.

#### Intraoperative Support

After the patient entered the room, we explained the surgical instruments and instructed the patient to cooperate well to ensure the smooth operation. Intraoperatively, a cardiac monitor was connected and all vital signs were closely monitored for 15 min/time. Control the intraoperative infusion volume <1,000 ml, and control the infusion temperature at about 36°C. The non-operative site was kept warm to prevent hypothermia and extremely wasted patients were given intraoperative warming blankets to maintain 37°C. Postoperative ECG was monitored continuously for 12 h, and pulse, blood pressure, temperature and respiratory changes were monitored.

#### Postoperative Support

Placed oxygen devices, suction devices and tracheotomy kits at the bedside, and reported to the physician as soon as the patient developed symptoms such as vocal cord paralysis, laryngeal edema, respiratory distress and asphyxia, and prepared for tracheotomy. Adequate postoperative pain relief can be achieved by providing patients with early intermittent neck ice, systematic pain education and, if necessary, non-steroidal analgesics, depending on the circumstances. After the patient was awake from anesthesia and the vital signs were stable, a small amount of water could be given, leg flexion and extension and turning activities could be performed in bed, and vocal exercises could be performed. Twelve hours after surgery, patients could eat semi-liquid diet and do some exercises around the bed; 24 h after surgery, they could gradually transition to general diet and walk in the corridor and rest area. On the first postoperative day, the patients were instructed to carry out functional neck exercises in a gradual manner, and the standard was that the patients did not show any discomfort symptoms, and at the same time, psychological guidance was provided to avoid patients being afraid to carry out exercises because they were worried about wound pain.

### Observation Indicators

The postoperative feeding time, hospitalization time and the first postoperative bed activity time and the 24 h postoperative pain level were compared between the two groups. The pain level was assessed by visual analog scoring (VAS), which was divided as 0–10, and the higher the score, the more severe the pain symptoms.

#### Nutritional Status

Before the intervention and on the second postoperative day of the patient, serum albumin (ALB), prealbumin (PA) and total protein (TP) levels were measured in the morning fasting venous blood of two groups of patients using a fully automated biochemical analyzer (Hitachi, Japan).

#### Negative Emotion

Before the intervention and on the second postoperative day, the patient's negative emotion was evaluated using the mental health test questionnaire (DCL-90), which includes 9 dimensions such as somatization, obsessive-compulsive symptoms, interpersonal relationship, sensitivity, depression, hostility, terror, paranoia, and psychoticism, with each dimension scored from 1 to 5.

#### Quality of Life

Patients were followed up at 3 months after discharge and their quality of life was evaluated using the General Quality of Life Inventory (GQOLI-74), which consists of four dimensions: physical, social, psychological and role, with a score of 100 out of 100.

#### Complications

The occurrence of postoperative complications, including hypocalcemia, choking on water, wound infection, numbness of hands and feet, and hoarseness, was recorded in both groups. Among them, serum calcium <8.7 mg·dL^−1^ was defined as hypocalcemia.

### Statistical Analysis

All data in this study were statistically analyzed using SPSS 20.0, and Prism 8.0 software was used to produce statistical graphs. The measures in the data were expressed as mean ± standard deviation (Mean,SD), and *t*-tests were performed between groups. The statistical data were expressed as rate (%), and the χ^2^ test was performed between groups. *p* < 0.05 was considered as a statistically significant difference.

## Results

### Comparison of the Base of 2 Groups

The results of the analysis showed that there were no significant differences between the control group and the experimental group in the general conditions of gender, age, tumor diameter, lesion site, body weight, preoperative albumin, total protein and prealbumin levels, NRS 2002 score, and operation time, and the base was comparable (*P* > 0.05) (Table 1).

### Comparison of Perioperative Indicators Between the 2 Groups

**Figure 1:**
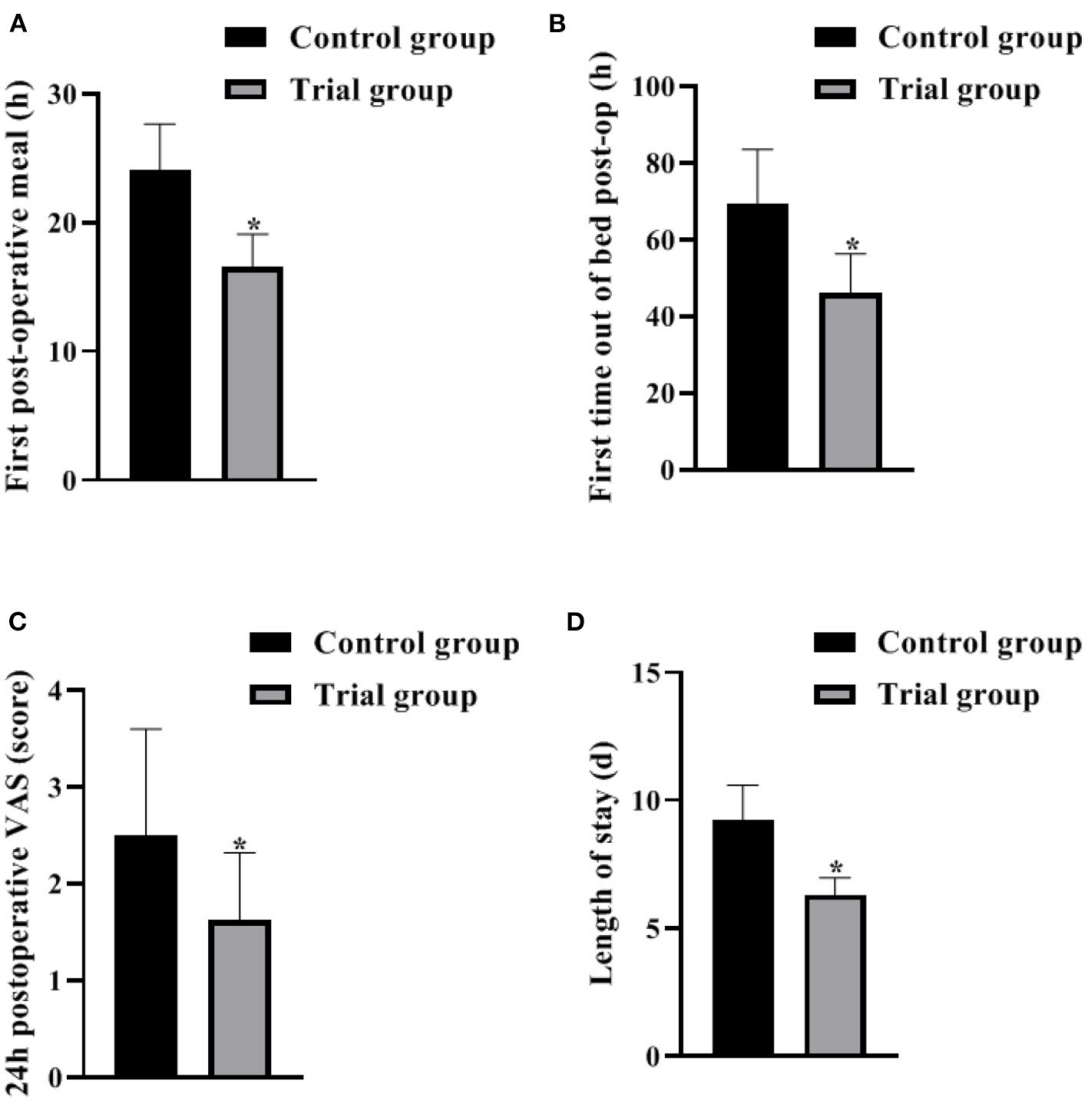
Comparison of perioperative indicators between the 2 groups (Mean, SD). The contents in **(A–D)** are the first postoperative meal, the first postoperative bed release, and the 24 h postoperative VAS score with the length of stay, respectively. The difference between the two groups *P < 0.05.

The results of the analysis showed that the first postoperative meal, the first postoperative bed release and the hospital stay in the trail group were significantly shorter than those in the control group (*P* < 0.05), suggesting that the FTS model based on nutritional support can promote the postoperative recovery of patients and shorten the hospital stay. In addition, the VAS score of the experimental group was lower than that of the control group at 24 h after surgery (*P* < 0.05), suggesting that the FTS model based on nutritional support can effectively prevent the occurrence of postoperative pain and reduce the degree of pain in patient (Figure 1).

### Nutritional Status of the 2 Groups

**Figure 2:**
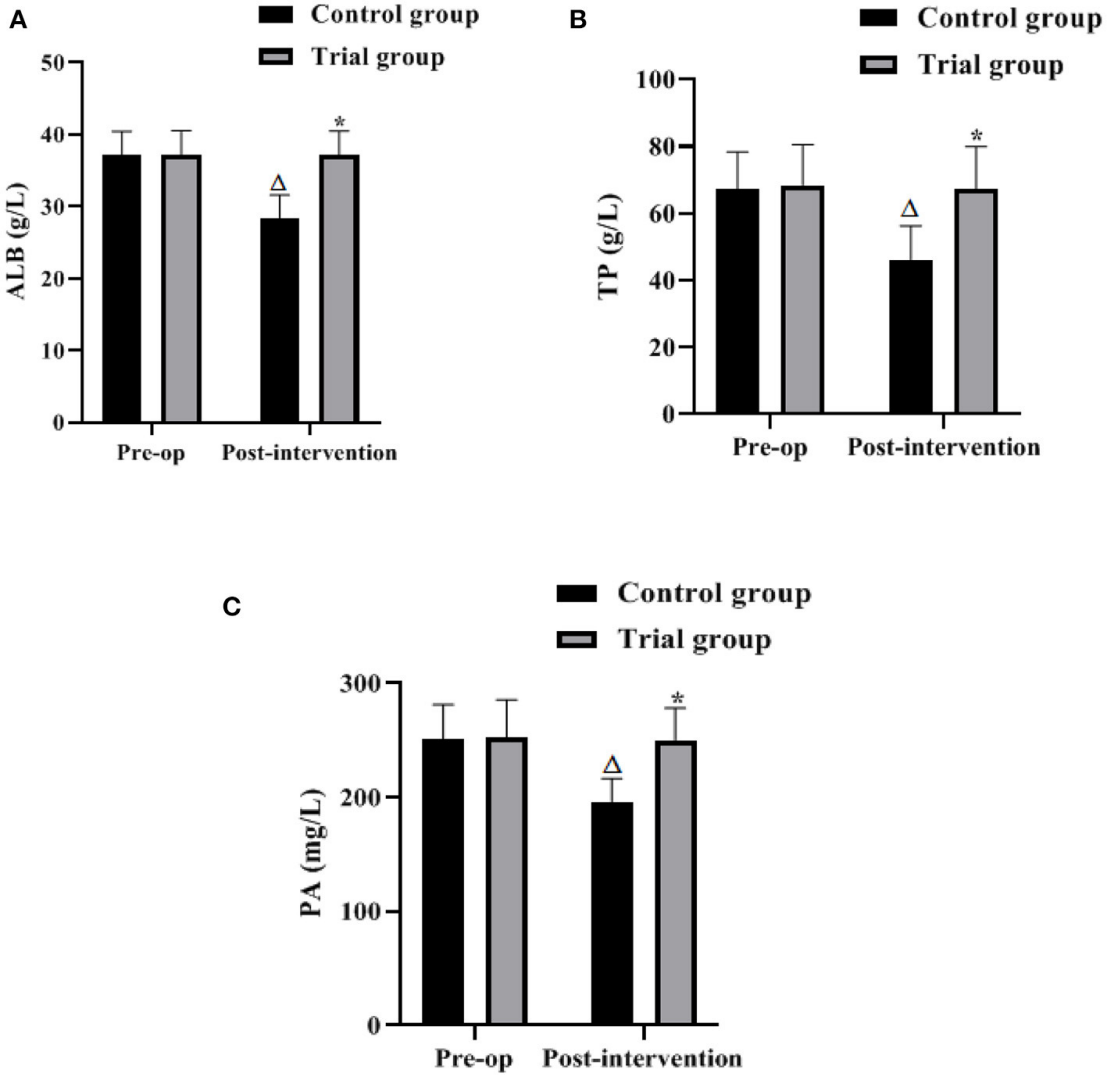
Nutritional status of the 2 groups (Mean, SD). The contents in **(A–C)** are ALB, TP, and PA levels, respectively. Difference from the same group preoperatively Δ*P* < 0.05; difference between the two groups *P < 0.05.

The results of the analysis showed that the preoperative ALB, TP and PA levels of the 2 groups did not differ significantly (*P* > 0.05). After the intervention, the levels of ALB, TP and PA in the control group were lower than those in the preoperative and concurrent experimental groups (*P* < 0.05), and the differences between the levels of ALB, TP and PA in the experimental group and the preoperative group were not significant (*P* > 0.05). It is suggested that the FTS model based on nutritional support can effectively reduce the occurrence of nutritional risks caused by various reasons in the perioperative period and improve the clinical outcomes of patients (Figure 2).

### Comparison of Negative Emotions Between the 2 Groups

**Figure 3:**
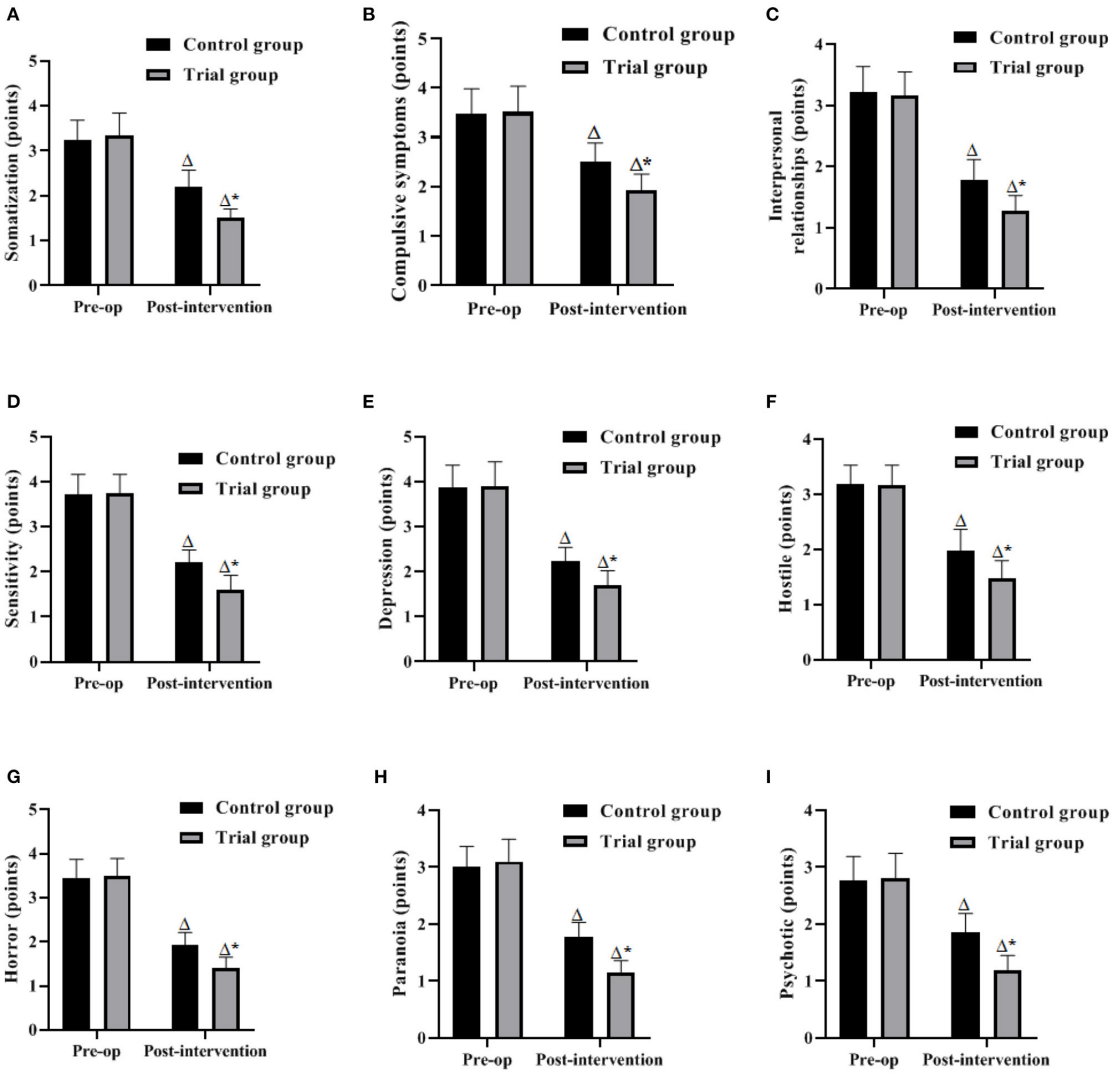
Comparison of negative emotions between the 2 groups (Mean, SD). The contents in **(A–I)** are somatization, obsessive-compulsive symptoms, interpersonal, sensitivity, depression, hostility, phobia, paranoia, and psychoticism, respectively. Difference from the same group preoperatively Δ*P* < 0.05; difference between the two groups *P < 0.05.

The results of the analysis showed that the differences in the scores of somatization, obsessive-compulsive symptoms, interpersonal relationship, sensitivity, depression, hostility, terror, paranoia, and psychoticism between the two groups before surgery were not significant (*P* > 0.05). After the intervention, the DCL-90 scores in both groups decreased significantly compared with the preoperative scores, and the scores of somatization, obsessive-compulsive symptoms, interpersonal relationship, sensitivity, depression, hostility, terror, paranoia, and psychoticism in the experimental group were lower than those in the control group (*P* < 0.05). It is suggested that the FTS model based on nutritional support can improve patients' negative emotions and is conducive to improving patients' treatment adherence (Figure 3).

### Comparison of the Quality of Life of the 2 Groups After 3 Months

**Figure 4:**
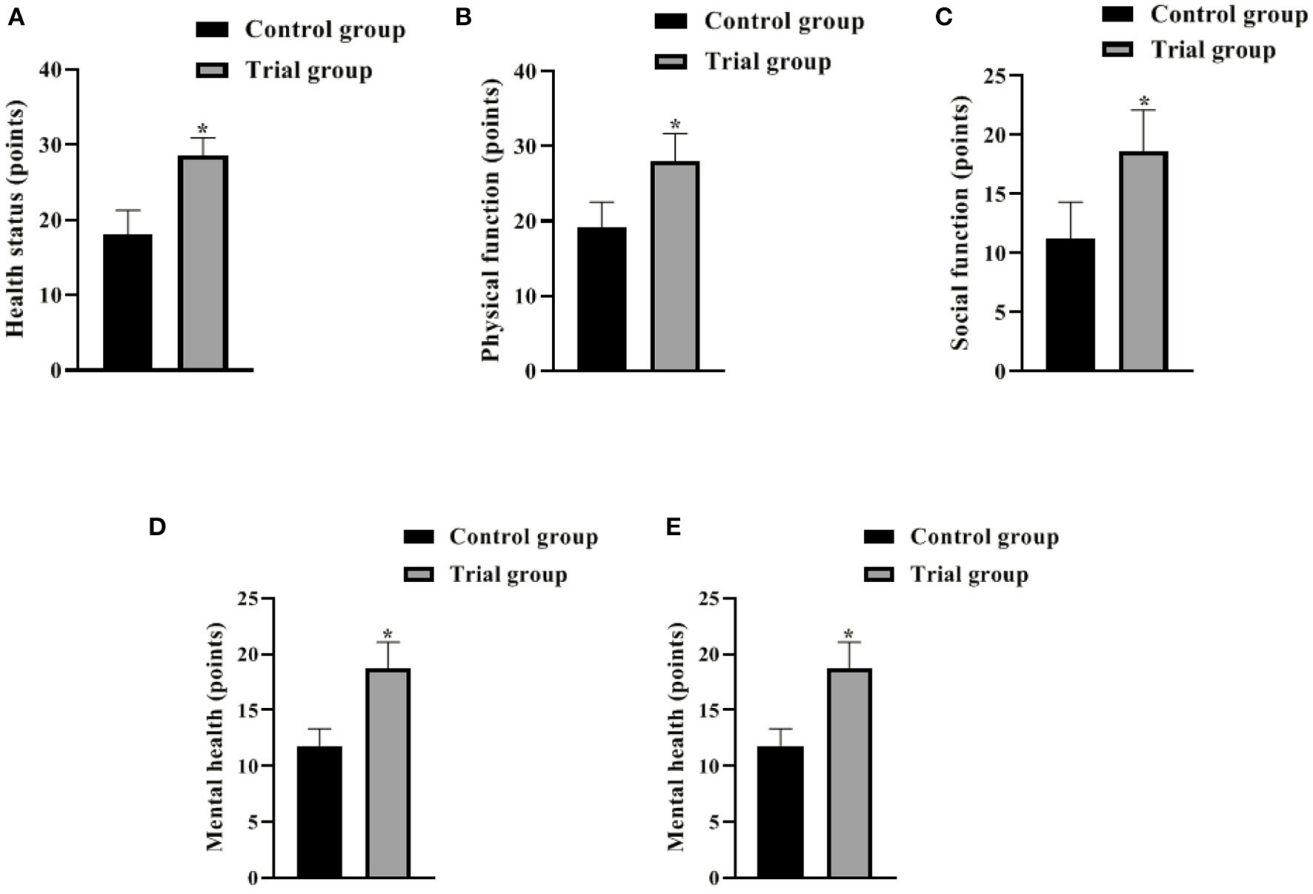
Comparison of the quality of life of the 2 groups after 3 months (Mean, SD). The contents in **(A–E)** are health status, physical function, social function, mental health and GQOLI-74 total score, respectively. The difference between the two groups *P < 0.05.

The analysis showed that the GQOLI-74 scores of health status, physical function, social function, and mental health as well as the total GQOLI-74 scores were higher in the experimental group than in the control group 3 months after surgery (*P* < 0.05). It is suggested that the FTS model based on nutritional support has a better effect on the improvement of patients' quality of life (Figure 4).

### Comparison of Complications Between the 2 Groups

In the control group, the incidence of hoarseness, choking on water, numbness of hands and feet, wound infection, hypocalcemia, and overall incidence were 5.00%, 7.50%, 10.00%, 5.00%, 2.50%, and 30.00%, respectively. In the trail group, the incidence of hoarseness, choking cough, numbness of hands and feet, traumatic infection, hypocalcemia, and overall incidence were 0.00%, 2.17%, 2.17%, 2.17%, 0.00%, and 6.52%, respectively. The analysis showed that the overall complication rate in the experimental group was lower than that in the control group (*P* < 0.05). It is suggested that the FTS model based on nutritional support can reduce the incidence of perioperative complications (Table 2).

**Table 2:** Comparison of complications between the 2 groups.

**Group**	**Hoarseness**	**Choking and coughing**	**Numbness of hands and feet**	**Wound infection**	**Hypocalcemia**	**Total**
Control group (*n* = 40)	2 (5.00)	3 (7.50)	4 (10.00)	2 (5.00)	1 (2.50)	12 (30.00)
Trail group (*n* = 46)	0 (0.00)	1 (2.17)	1 (2.17)	1 (2.17)	0 (0.00)	3 (6.52)
χ^2^ value	-	-	-	-	-	8.190
*P-*value	-	-	-	-	-	0.004

## Discussion

Thyroid cancer is a malignant tumor originating from thyroid follicular epithelial cells. It mainly presents as painless neck nodules or masses, it is more common in young adults, and the incidence in women is about 2–4 times that of men. Currently, the disease is mainly treated by surgery, radionuclide therapy and endocrine therapy (12, 13). In recent years, with the continuous development and improvement of minimally invasive techniques, lumpectomy also plays an important role in the treatment of thyroid cancer, with the advantages of less trauma, less bleeding, faster recovery and better aesthetics (14). However, radical thyroidectomy is difficult and prone to many postoperative complications. In addition, due to the pathological consumption of thyroid cancer, dysphagia symptoms, surgery and inflammation, a large amount of proteins and fats are consumed in the body, and patients may suffer from malnutrition status (15, 16). One study (17) showed that patients with nutritional risk have a significantly higher incidence of postoperative infectious complications and a significantly longer hospital stay, so clinical workers need to pay attention to nutritional screening of surgical patients and the prognostic impact of nutritional risk on surgery. The results showed that the postoperative albumin, total protein, prealbumin levels, GQOLI-74 scores and total scores of the test group were higher than those of the control group; the first postoperative meal, first time out of bed and hospital stay were shorter than those of the control group; the postoperative 24 h VAS score, postoperative DCL-90 scores and total postoperative complication rate were lower than those of the control group. The present results confirm that a FTS model based on nutritional support significantly improves the clinical outcomes of patients undergoing endoscopic radical thyroidectomy, promotes patient recovery, and contributes to the improvement of patients' postoperative quality of life.

FTS refers to the application of various methods that have been proven effective by evidence-based medicine before, during, and after surgery to reduce surgical stress and complications and accelerate postoperative recovery (18, 19). FTS as a new treatment concept has achieved great success in the fields of general surgery, gynecology, and urology, and in recent years, FTS has gradually been widely used in the clinical treatment of thyroid cancer, resulting in faster postoperative recovery and significantly shorter hospitalization. The length of hospitalization has been significantly shortened (20, 21). In this study, a rapid perioperative rehabilitation model suitable for lumpectomy for radical thyroid cancer was developed to address the special characteristics of thyroid cancer, which mainly includes active and effective health education, dietary management and postural training before surgery, intraoperative hypothermia prevention, restrictive fluid rehydration and placement of surgical positions, and comprehensive perioperative management measures such as multimodal analgesia, early feeding, early activity and early functional exercise after surgery. The above measures obviously reduced the interference with normal body functions, and the intestinal functions of thyroid cancer patients were not significantly affected. Based on the fact that enteral nutritional support is more physiological than parenteral nutritional support and more beneficial to maintain the structural and functional integrity of intestinal mucosal cells, enteral nutritional support should be preferred (22, 23). Studies (24, 25) have shown that high protein intake increases intestinal absorption of calcium, stimulates insulin-like growth factor secretion, increases BMI, enhances muscle strength, and reduces the incidence of complications. Reasonable and standardized perioperative nutritional support can promote protein synthesis and tissue healing, improve surgical tolerance, reduce the damage caused by stress, inhibit the reduction of the body's immune function, control the inflammatory response, and promote patient recovery (26).

Improved nutrition may lead to earlier time to floor in patients undergoing radical lumpectomy for thyroid cancer, while early activity may promote gastrointestinal motility as well as reduce the incidence of perioperative complications. This study confirms that the FTS model based on nutritional support can effectively reduce the incidence of postoperative complications, shorten the number of hospital days, and promote postoperative recovery in patients undergoing lumpectomy for radical thyroid cancer, thereby improving clinical outcomes.

## Data Availability Statement

The original contributions presented in the study are included in the article/supplementary material, further inquiries can be directed to the corresponding author.

## Ethics Statement

This study was approved through the Hospital Ethics Committee. The patients/participants provided their written informed consent to participate in this study.

## Author Contributions

FQ and CX are the mainly responsible for the writing of the article. HB is mainly responsible for research design. FQ is mainly responsible for data analysis. LY and HL are responsible for the guidance of the entire research. All authors contributed to the article and approved the submitted version.

## Conflict of Interest

The authors declare that the research was conducted in the absence of any commercial or financial relationships that could be construed as a potential conflict of interest.

## Publisher's Note

All claims expressed in this article are solely those of the authors and do not necessarily represent those of their affiliated organizations, or those of the publisher, the editors and the reviewers. Any product that may be evaluated in this article, or claim that may be made by its manufacturer, is not guaranteed or endorsed by the publisher.
